# Comparison of Succinylcholine, Rocuronium, and Rocuronium with Magnesium on Time of Onset of Paralysis in Adult Patients Undergoing Rapid Sequence Induction: A Double Blinded Randomised Control Trial

**DOI:** 10.4274/TJAR.2025.251886

**Published:** 2025-12-22

**Authors:** George Paul, Shagufta Naaz, Umesh Kumar Bhadani, Nishant Sahay, Rajnish Kumar, Satish Kumar

**Affiliations:** 1 St. John’s National Academy of Health Sciences, Bangalore; All India Institute of Medical Sciences, Patna, Department of Anaesthesiology, Patna, Bihar, India; 2 All India Institute of Medical Sciences, Patna, Department of Anaesthesiology, Patna, Bihar, India

**Keywords:** Intubation, laryngoscopy, muscle relaxation, magnesium sulphate, rocuronium, succinylcholine, vocal cords

## Abstract

**Objective:**

We compared magnesium sulphate pre-treatment with rocuronium at a dose of 0.9 mg kg^-1^ to the standard succinylcholine (1 mg kg^-1^) in rapid sequence induction to see if this combination had an onset of paralysis comparable to succinylcholine.

**Methods:**

This was a prospective, single-centre, double-blinded, parallel-arm, randomized controlled trial on patients aged 18-60 years, either sex, the American Society of Anesthesiologists I and II. Patients received a 100 mL normal saline infusion followed by either succinylcholine at 1 mg kg^-1^ (Group S), or rocuronium 0.9 mg kg^-1^ (Group R), or a 100 mL normal saline infusion containing magnesium sulphate 60 mg kg^-1^, followed by rocuronium 0.9 mg kg^-1^ (Group MgR). The primary outcome was the time of onset of paralysis evidenced by fading of train-of-four (TOF). Secondary outcomes were the intubation conditions, and the laryngoscopy response.

**Results:**

Data from 135 patients showed TOF fading times differed significantly across the groups, with Group S showing a median (*interquartile range*-IQR) of 65 (61-70) seconds, Group R 102 (98-108) seconds, and Group MgR 82 (79-85) seconds (*P* < 0.001). The ease of laryngoscopy and response to cuff inflation showed no significant difference (*P*=1.000). Analysis of the position of the vocal cords suggested a significant difference (*P* < 0.001). Finally, the total intubating conditions indicated a significant difference among the groups (*P* < 0.001), favouring Group MgR for excellent intubating conditions.

**Conclusion:**

The onset of action was significantly faster with succinylcholine than with magnesium sulphate-rocuronium. Nevertheless, it was significantly faster with magnesium sulphate-rocuronium than with rocuronium alone. However, the intubation conditions were better when magnesium was added to rocuronium.

**Main Points**

• The onset of action is significantly faster with succinylcholine than that with rocuronium at a dosage of 0.9 mg kg^-1^ alone or with magnesium sulphate added.

• However, the muscle relaxation and intubation conditions are better when magnesium is added to rocuronium in comparison with succinylcholine or rocuronium alone.

• Magnesium sulphate addition provided stable hemodynamic condition, blunting the laryngoscopy response.

## Introduction

Rapid sequence induction (RSI) is usually performed in the emergency department or in specific conditions that require prompt and secure control of the patient’s airway.^1^ It is commonly used when the patient has a high risk of aspiration, such as when the patient has a full stomach or is pregnant.^2^ The choice of drugs is critical, with succinylcholine being the preferred agent due to its rapid onset and short duration, facilitating quick intubation and reducing the risk of aspiration. The recommended dose for RSI is 1-1.5 mg kg^-1^, which gives an onset time of approximately 45-60 seconds.^3^ It even provides excellent intubation conditions, which makes it particularly valuable in emergencies and thereby increases the chances of first-pass success of endotracheal intubation.^4^

However, succinylcholine’s adverse effects, such as bradycardia, arrhythmias, and hyperkalaemia, among others, necessitate alternatives in certain patient groups.^5^ When succinylcholine is contraindicated or not preferred, rocuronium is commonly used as an alternative non-depolarising muscle relaxant in RSI.^6^ However, to achieve a similar speed of onset as succinylcholine, along with excellent intubating conditions, a high dose of rocuronium of 1.2 mg kg^-1^ is typically required.^7^ One of the major drawbacks of using a high-dose non-depolarizing muscle relaxant is its prolonged duration of action. This extended effect can be mitigated by sugammadex; however, its high cost is a limitation for its use.^8^

Magnesium sulphate has been extensively studied for its uses in the perioperative period. Known for its perioperative benefits, including reducing the onset time of non-depolarising muscle relaxants and enhancing intubation conditions, magnesium sulphate presents a potential solution.^9^ It also offers cardioprotective effects, hemodynamic stability, and neuroprotection. Nalini et al.^10^ showed that pretreatment of magnesium sulphate accelerates neuromuscular block as compared to vecuronium with or without priming. Previous research and studies primarily focused on the impact of pretreatment with magnesium on rocuronium in its standard dose (0.6 mg kg^-1^) in assessing intubating conditions.^11^ However, there is still limited evidence regarding the onset time of paralysis of rocuronium at a slightly higher dose of 0.9 mg kg^-1^, with magnesium sulphate and its use in RSI.

Through this study, we aim to address the gap in evidence by comparing the effects of magnesium sulphate pre-treatment with rocuronium at a dose of 0.9 mg kg^-1^ to the standard treatment with succinylcholine and rocuronium without magnesium sulphate pre-treatment in RSI. We hypothesized that 0.9 mg kg^-1^ rocuronium when preceded by magnesium sulphate could achieve comparable onset times and intubating conditions as succinylcholine at 1 mg kg^-1^. We have tried to find out if this combination can provide an onset of paralysis comparable to succinylcholine, potentially offering a safer alternative for patients in whom succinylcholine is contraindicated. This research could refine RSI practice by providing an alternative to succinylcholine, thereby enhancing patient safety and care in critical situations.

## Methods

The present study was a double-blinded randomized controlled trial and was carried out in the in a tertiary institute, in compliance with the standards outlined in the World Medical Association Declaration of Helsinki after receiving the All India Institute of Medical Sciences, Patna, Institutional Ethics Committee’s approval (approval no.: AIIMS/Pat/IEC/PGTh/Jan21/52, and dated: 29^th^ December 2021). Trial Registration was completed (www.ctri.nic.in) with registration no.: CTRI/2022/02/040528, dated February 23, 2022. Patients undergoing elective surgery under general anaesthesia, with an anticipated duration of surgery >60 minutes, of American Society of Anesthesiologists (ASA) physical status I and II, and age group 18-60 years, belonging to either sex with body mass index (BMI) between 18 kg m^2-1^ and 28 kg m^2-1^ were included in the study. The exclusion criteria were patients with contraindications to any of the study drugs, patients with neuromuscular diseases, electrolyte imbalances, anticipated difficult intubation or mask ventilation, and pregnancy.

Patients were allocated to one of three groups with a computer-generated random number table. The study groups were defined as follows:

- Group S received a 100 mL normal saline infusion over 10 minutes followed by succinylcholine at 1 mg kg^-1^.

- Group R received a 100 mL normal saline infusion over 10 minutes followed by rocuronium at 0.9 mg kg^-1^.

- The MgR group received a 100 mL normal saline infusion with magnesium sulphate 60 mg kg^-1^ over 10 minutes, followed by rocuronium of 0.9 mg kg^-1^.

Block randomization with a fixed block size of nine was utilized to assign patients to their respective groups. Concealment of allocation was achieved using the opaque sealed envelope method. The envelopes, prepared by a statistician, were handed over to the anaesthesia team by an operating room technician. The anaesthetist in the operating room opened the envelope to reveal the allocated anaesthesia technique.

The patient was unaware of the infusion given before induction. This involved administering saline or magnesium sulphate in a 100 mL 0.9% normal saline solution, 10 minutes before induction. Secondly, to mitigate the potential observer bias due to succinylcholine-induced fasciculations, the expert anaesthesiologist, responsible for intubation and assessing the intubating conditions, was called into the operating room once the fasciculations had subsided (at least 45 seconds after the injection of the drug). Both succinylcholine and rocuronium were diluted to 10 mL and prepared by the operating room personnel not involved in the study. This ensured that the study personnel remained unaware of the specific drugs being administered. Additionally, another anaesthesiologist, who was not directly involved in the study, noted down the train-of-four (TOF) values, while a junior anaesthesiologist monitored and recorded the vital signs of the patient.

Written informed consent was obtained from all participants for the study. A detailed pre-anaesthetic check-up was conducted, and informed written consent was obtained. Clinical characteristics such as age, sex, height and weight, and BMI were recorded. In the operating room, standard ASA monitoring devices were attached: pulse oximeter, non-invasive blood pressure, electrocardiogram, temperature monitor. Neuromuscular monitoring was performed using Drager® Infinity® Trident® neuromuscular transmission (NMT) SmartPod®. NMT study was done on the ulnar nerve to adductor pollicis muscle group. Group MgR patients were given 60 mg kg^-1^ magnesium sulphate infusion in 100 mL of 0.9% normal saline over a period of 10 minutes, whereas patients in the other two groups received 100 mL of 0.9% normal saline infusion. Pre-oxygenation was then performed for a period of 3 minutes, to achieve FeO_2_ >91%, followed by IV fentanyl 2 µg kg^-1^ and propofol 2 mg kg^-1^. After induction, according to the randomization, patients were given the respective muscle relaxant. Onset time was then calculated from the end of the muscle relaxant injection to the disappearance of three TOF twitches for rocuronium and the complete disappearance of TOF for succinylcholine. Intubation was then done by an expert anaesthesiologist (with more than 5 years of experience) who was not a part of the study.

Intubation scores (using ease of laryngoscopy, the position of vocal cords, and the reaction to cuff inflation) and the laryngoscopy response (by noting the hemodynamic parameters) were assessed. All the vitals of the patients [heart rate (HR), systolic blood pressure (SBP), diastolic blood pressure (DBP), and SpO_2_] were recorded every 3 minutes for the first 10 minutes after the injection of the muscle relaxant. The rest of the surgery continued according to the standard protocol followed by the institute.

TOF measurement settings were as follows:

• Mode - TOF monitoring,

• Measurement interval - 10 seconds,

• Pulse width - 100 microseconds.

The intubation score was based on 3 parameters:

• Ease of laryngoscopy - Easy/Fair/Difficult,

• Position of vocal cords - Abducted/Intermediate/Adducted,

• Response to cuff inflation - None/Slight/Vigorous,

Total intubating score - Excellent/Good/Poor.

Laryngoscopy-easy (jaw relaxed, no resistance to blade insertion), fair (jaw not fully relaxed, slight resistance to blade insertion), difficult (poor jaw relaxation, active resistance of the patient to laryngoscopy)

Response to cuff inflation - Slight (one to two weak contractions and/or movement for <5s),

Vigorous (more than 2 contractions and/or movement for >5s)

Total intubation score - Excellent (all qualities are excellent), Good (all qualities are either excellent or good) and Poor (presence of a single quality listed under “poor”)

Any specific complications or adverse events that occurred were also noted.

### Primary Outcome

The primary outcome was to compare the time of onset of paralysis as evidenced by fading of TOF between rocuronium (0.9 mg kg^-1^) with magnesium and succinylcholine(1 mg kg^-1^), by keeping rocuronium (0.9 mg kg^-1^) as a control group.

### Secondary Outcomes

Secondary outcomes were to compare the intubation conditions between the muscle relaxants and to assess the laryngoscopy response using hemodynamic parameters measured every 3 minutes or until 10 minutes after injection of the muscle relaxant.

To calculate the sample size, a previous study^12^ was taken into consideration, and an online calculator was used to determine the minimum number of patients required for each group. Taking alpha as 0.05 and beta as 0.2, and using the difference between the two means and the expected pooled standard deviation, the sample size was calculated to be at least 39 patients for each group. Considering the dropout rate of 10%, a total of 45 patients in each group were enrolled. The control group of the same sample size was added. Therefore, the total sample size was 135.

### Statistical Analysis

The collected data were analysed using Jamovi 2.3.26.0, statistical software, Sydney, Australia (open source). Results were expressed as mean ± standard deviation (SD), n (number), median, or interquartile range (IQR) where appropriate. Data were evaluated to determine whether a normal distribution exists, and the central tendency was appropriately represented. Fisher’s exact or chi-square tests were used for categorical variables, and one-way analysis of variance (ANOVA) and repeated measures ANOVA were used for continuous variables. A *P* value < 0.05 was considered statistically significant. For post-hoc analysis, Bonferroni correction was implemented. As there were three groups, alpha error was corrected for intergroup comparison. The new alpha level would be 0.0167 (0.05/3).

## Results

**Figure 1:**
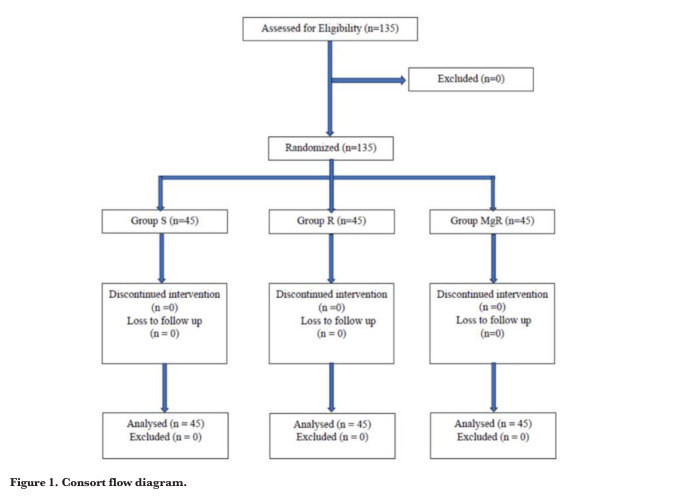
Consort flow diagram.

One hundred and thirty-five patients were assessed for eligibility, randomized, and allocated to one of the three groups of forty-five each (Figure 1). None of them were lost to follow-up, and the results of analysis have been described below.

### Demographic and Clinical Characteristics of the Patients

The demographic analysis of this study’s participants showed the normality of age, weight, height, and BMI, which was confirmed using Q-Q plots. The mean age, BMI, height, and weight across the groups did not show a significant difference among the groups. These findings collectively indicate that there was a uniform distribution of demographic variables across the groups, ensuring a balanced comparison of the study’s outcomes (Table 1). Gender distribution has been represented in Table 1. Overall, the study’s total sample size had a female representation of 54.8% and a male representation of 45.2%.

### Primary Outcome

**Figure 2:**
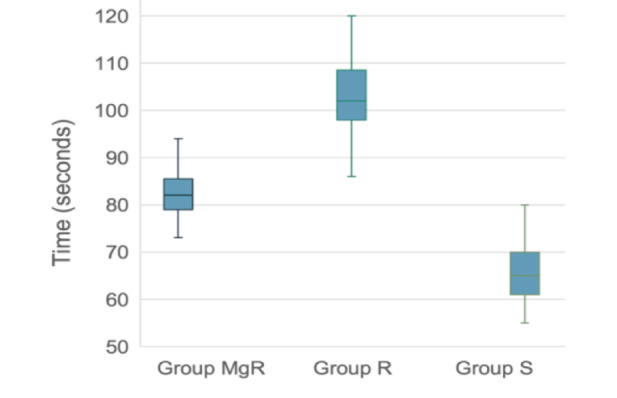
Box-Whisker plot of Train of Four among three groups.

The primary outcome, i.e., the TOF fading/disappearance times, differed significantly across the groups, with Group S showing a median (IQR) of 65 (61-70) seconds, Group R showing a median (IQR) of 102 (98-108) seconds, and Group MgR showing a median (IQR) of 82 (79-85) seconds (Figure 2). One-way ANOVA (non-parametric Kruskal-Wallis) test showed a χ² value of 110 and an overall *P *value of < 0.001. Post-hoc analysis using Dwass-Steel-Critchlow-Fligner pairwise comparisons showed that there is a significant difference between each pair of groups with a *P* value of < 0.001. Thus, the onset time of action of the succinylcholine group was significantly faster than that of both other groups. However, the onset of action of the group that received magnesium sulphate pretreatment with rocuronium was significantly faster than the group with rocuronium alone.

### Secondary Outcomes

In terms of ease of laryngoscopy (Table 2), all groups performed well, with most cases being classified as easy. A *P* value of 1.000 indicated no significant difference in the ease of laryngoscopy between groups.

The position of vocal cords was predominantly open in all groups (Table 2). Fisher’s exact test was performed as the expected count was less than 5, which showed a *P* value of < 0.001, suggesting that there is a significant difference among the three groups. Cramer V test for association showed a value of 0.361, which indicates a strong association. A *P* value of less than 0.001 suggested a significant difference, particularly indicating that Group R has more intermediate positions.

Regarding the response to cuff inflation, all groups showed no response in most cases, with a *P* value of 1.000.

Finally, the total intubating conditions were excellent in a high percentage of cases across all groups. A chi-square test of independence was performed for the total intubating conditions in the three groups. The expected frequencies in all the cells were greater than five. Results showed a χ² value of 17.9 and a *P* value of < 0.001, suggesting that there is a significant difference between the three groups with a χ² value in favour of Group MgR. Cramer V test for association showed a value of 0.364, which is suggestive of a moderate level of association.

Table 3 represents the repeated measures ANOVA, which showed significant changes in HR, systolic blood SBP, and DBP in the initial 10 minutes post-administration of muscle relaxants. Violations of sphericity were corrected using Greenhouse-Geisser adjustments (ε values of 0.894 for HR, 0.744 for SBP, and 0.873 for DBP). The findings were statistically significant, with changes in HR (F = 31.9, *P *< 0.001), SBP (F = 12.63, *P* < 0.001), and DBP (F = 3.61, *P*=0.003), demonstrating notable variations in these cardiovascular parameters over time.

Post hoc analyses using Bonferroni adjustments revealed significant cardiovascular differences among the study groups (Table 4). Heart rate, and systolic blood pressure, significantly increased when comparing Group S to MgR and Group R to MgR, while no similar differences were observed between Group S and R. DBP was significantly higher in Group S than in Group MgR, but remained comparable between Group S and R, and Group R and MgR.

## Discussion

In our study, succinylcholine showed a significantly faster onset time for muscle paralysis than both rocuronium groups, with and without magnesium sulphate. The median (IQR) TOF fading/disappearance times were notably different, with succinylcholine at 65 (61-70) seconds, rocuronium alone at 102 (98-108) seconds, and rocuronium with magnesium sulphate at 82 (79-85) seconds. These results align with the known rapid action of succinylcholine.^7^ However, adding magnesium sulphate to rocuronium did improve intubating conditions significantly, achieving excellent conditions in all subjects within the MgR group. Magnesium sulphate’s mechanism of action at the neuromuscular junction, which involves the inhibition of calcium and acetylcholine release, might have contributed to the reduction in the onset time of neuromuscular relaxants when comparing it with rocuronium used alone.

Our study reported a 20% reduction in onset time with magnesium sulphate pre-treatment, as the median-IQR of the TOF value was 102 (98-108) seconds in group R and 82 (79-85) seconds in group MgR. A study done by Czarnetzki et al.^13^ on the effect of magnesium sulphate pretreatment 15 minutes, prior to the standard dose of rocuronium (0.6 mg kg^-1^) showed that the decrease was about 35%. This observed variation in the decrease in onset time could be attributed to the time required for the full effect of magnesium sulphate to manifest, which might not have been realized in our study’s protocol, where magnesium sulphate was administered 10 minutes before the start of induction.

In contrast, when comparing the time of onset of muscle paralysis between succinylcholine (1 mg kg^-1^) and a high dose of rocuronium (1.2 mg kg^-1^), the high dose of rocuronium showed that the onset time was comparable between the succinylcholine group and the combination of rocuronium and magnesium. This is not in accordance with our findings.^12^ The divergent outcomes might be the result of higher rocuronium doses used in the comparative study, which warrants cautious interpretation due to the limited sample size.

A prospective study conducted by Han et al.^14^ showed that administering 1.2 mg kg^-1^ of rocuronium did not significantly hasten the onset of neuromuscular block compared to the 0.9 mg kg^-1^ dose, indicating a ceiling effect where the time required for rocuronium to reach the neuromuscular junction may be the limiting factor. However, the duration of the neuromuscular block was extended significantly with the 1.2 mg kg^-1^ dose compared to the 0.9 mg kg^-1^ dose.^14^

Most of the studies have compared intubating conditions. A recent study by Czarnetzki et al.^15^ showed that there were comparable intubating conditions using the gold standard succinylcholine (1 mg kg^-1^) and magnesium sulphate with rocuronium (0.6 mg kg^-1^). In our study, a dose of rocuronium of 0.9 mg kg^-1^ along with pretreatment of magnesium sulphate resulted in significantly better intubating conditions when compared with succinylcholine of 1 mg kg^-1^. Thus, our study demonstrated that administration of magnesium as a pretreatment, followed by rocuronium at a comparatively lower dose of 0.9 mg kg^-1^ (compared to the standard 1.2 mg kg^-1^) yielded more favourable conditions for intubation, compared to the traditional and widely accepted succinylcholine. The difference in our finding compared to the aforementioned study might be due to the different doses of rocuronium used. Czarnetzki et al.^15^ did not investigate the onset time of action between succinylcholine and rocuronium. They found that the rate of excellent intubating conditions was significantly higher in women than in men after receiving magnesium-rocuronium. However, we did not investigate this aspect of gender association.

Upon thoroughly analyzing the variables employed to assess the overall intubating conditions, it was observed that the succinylcholine group had more subjects with intermediate positioning of the vocal cords compared to the group receiving magnesium sulphate with rocuronium. This finding implies that the combination of magnesium sulphate and rocuronium potentially reduces the resistance of laryngeal muscles to non-depolarizing muscle relaxants.^16^ This suggests that magnesium may have a beneficial effect in enhancing the relaxation of laryngeal muscles when used in conjunction with rocuronium during intubation procedures. Therefore, this combination of rocuronium and magnesium sulphate has the potential to enhance the intubating conditions further and improve overall intubation success.

Our study also revealed that magnesium sulphate provided haemodynamic stability, blunted the laryngoscopy response, and reduced SBP and HR in the initial 10 minutes post-administration when compared with the other groups. These findings are consistent with other studies that have reported similar hemodynamic stability with magnesium sulfate.^17^ Czarnetzki et al.^15^ observed no significant difference between groups for systolic and diastolic blood pressures; however, mean HR was significantly higher in the magnesium sulphate-rocuronium group. Furthermore, we did not quantitatively measure the prolongation of the duration of action of rocuronium with magnesium pre-treatment. However, it was evident that magnesium sulphate extended the duration of action compared to rocuronium alone, likely due to its inhibitory effect on acetylcholine released at the motor endplate.

While succinylcholine does result in paralysis more quickly according to statistics, the actual significance of this slightly faster action is uncertain when compared to the improved conditions for intubation provided by the combination of magnesium and rocuronium. This benefit becomes even more important in the case of obese patients, who may have difficulties with intubation due to their anatomy and increased adipose tissue. Therefore, it is crucial to ensure that muscle relaxation and intubation conditions are optimal for the successful placement of the endotracheal tube. The study suggests that using magnesium sulphate with rocuronium at a dosage of 0.9 mg kg^-1^, could be an effective replacement for the usual succinylcholine at 1 mg kg^-1^ RSI.

Some adverse effects due to the drugs were noted. Post-surgery, a few subjects in the succinylcholine group reported having slight muscle pain, which was very much self-limiting and under control by the use of non-steroidal anti-inflammatory drugs. In group MgR, during the administration of magnesium sulphate as a pretreatment, a small number of patients reported experiencing pain at the injection site. Some individuals also reported mild dizziness while the infusion was ongoing. All these side effects were very mild and may not even be considered medically significant. One serious adverse effect that was noted is that one patient in the magnesium group developed bradycardia, which was managed with appropriate medical intervention (immediately corrected by the administration of atropine).

### Study Limitations

The study’s limitations include a small sample size of 45 patients per group, which challenges the generalizability of the findings, and the potential for a ±10 seconds error in measuring the time onset which was the minimum settings of the monitoring equipment. Additionally, being a single-center study limits the applicability of the results to a broader population.

## Conclusion

The onset of action was significantly faster with succinylcholine than with rocuronium at a dosage of 0.9 mg kg^-1^ alone or with magnesium sulphate added. Nevertheless, rocuronium with magnesium sulphate had a significantly faster onset of action compared to rocuronium alone. However, the muscle relaxation and intubation conditions were better when magnesium was added to rocuronium in comparison with succinylcholine or rocuronium alone.

Based on the findings of this study, we recommend the following:

1. Conducting larger studies involving multiple centres to validate and strengthen the evidence which supports the use of magnesium sulphate with rocuronium in RSI.

2. Further evaluating the clinical impact of succinylcholine, considering the minimal observed difference.

3. Monitoring closely for potential side effects such as low blood pressure and slow HR when administering magnesium.

## Ethics

**Ethics Committee Approval:** Ethical approval was obtained from the All India Institute of Medical Sciences, Patna, Institutional Ethics Committee (approval no.: AIIMS/Pat/IEC/PGTh/Jan21/52, and dated: 29^th^ December 2021).

**Informed Consent:** Written informed consent was obtained from all participants for the study.

## Figures and Tables

**Table 1. Demographic Characteristics of the Participants (Mean ± SD, n or Percent):** 

**Demographic variables**	**Group S** **(Mean ± SD)**	**Group R** **(Mean ± SD)**	**Group MgR(Mean ± SD)**	***P* value**
Age (years)	41.3±12.1	35.7±14.1	37.5±14.1	0.119^#^
Weight (kg)	59.7±8.01	57.2±7.86	59.4±7.37	0.253^#^
Height (cm)	159±6.73	159±7.28	159±6.36	0.941^#^
BMI (kg m^2-1^)	23.7±3.14	22.6±2.98	23.7±2.78	0.166^#^
Male:Female n (%)	20 (14.8%):25 (18.5%)	18 (13.3%):27 (20.0%)	23 (17.0%):22 (16.3%)	0.567^##^ (χ²=1.14)

^#^ANOVA; ^##^chi-square test; *P* < 0.05 considered as significant.

ANOVA, analysis of variance; BMI, body mass index; SD, standard deviation.

**Table 2. Clinical Parameters of the Participants:** 

**Clinical parameter**	**Group S (n = 45)**	**Group R (n = 45)**	**Group MgR (n = 45)**	*P* value*
**Ease of laryngoscopy**				
Easy (n)	44	44	45	1.000
Fair (n)	1	1	0	
Difficult (n)	0	0	0	
**Position of vocal cords**				
Abducted (n)	42	33	45	<0.001**
Intermediate (n)	3	12	0	
Adducted (n)	0	0	0	
**Response to cuff inflation**				
None (n)	45	44	45	1.000
Slight (n)	0	1	0	
Vigorous(n)	0	0	0	
**Total intubating condition**				
Excellent (n)	41	32	45	χ² =17.9 <0.001**
Good (n)	4	13	0	
Poor (n)	0	0	0	

*Fisher’s exact test; **P < 0.05; n, number.

**Table 3. Hemodynamic Variables at different Intervals Among Three Groups (Mean ± SD):** 

**Value**	**Interval**	**Group S** **(Mean ± SD)**	**Group R** **(Mean ± SD)**	**Group MgR(Mean ± SD)**	**F value**	***P* value^#^**
**HR**	Pulse 1	80.8±10.80	82.8±13.63	81.4±9.28	31.9	<0.001*
	Pulse 4	91.7±9.69	93.5±12.79	77.9±10.88		
	Pulse 7	93.4±9.74	94.0±13.86	78.7±10.48		
	Pulse 10	92.0±9.42	90.8±12.01	80.2±9.40		
**SBP**	SBP 1	126±12.26	123±13.89	127±10.93	12.63	<0.001*
	SBP 4	135±15.93	133±18.79	120±10.54		
	SBP 7	134±12.75	132±15.44	123±10.05		
	SBP 10	132±11.53	128±14.12	123±7.82		
**DBP**	DBP 1	77.9±10.2	79.3±9.43	75.8±10.08	3.58	0.003*
	DBP 4	80.1±12.45	78.3±12.87	76.3±9.53		
	DBP 7	83.9±9.72	83.3±10.47	75.6±8.55		
	DBP 10	83.2±8.54	80.6±10.59	76.2±8.61		

^#^Repeated measures ANOVA, *P < 0.05.

ANOVA, analysis of variance; HR, heart rate; SBP, systolic blood pressure; DBP, diastolic blood pressure.

**Table 4. Post-hoc Analysis Between the Hemodynamic Variables:** 

**Parameter**	**Comparison**	**Mean difference**	**SE**	**df**	***P* value#**
**HR**	S-R	-0.778	2.18	132	1.000
	S-MgR	9.900	2.18	132	<0.001*
	R-MgR	10.768	2.18	132	<0.001*
**SBP**	S-R	2.82	2.36	132	0.701
	S-MgR	8.59	2.36	132	0.001*
	R-MgR	5.77	2.36	132	0.047
**DBP**	S-R	0.917	1.84	131	1.000
	S-MgR	5.409	1.85	131	0.012*
	R-MgR	4.492	1.85	131	0.050

#Bonferroni correction as post-hoc analysis, *P value < 0.0167 considered statistically significant.

HR, heart rate; SBP, systolic blood pressure; DBP, diastolic blood pressure; SE, standard error; df, degrees of freedom.
